# The Place of Meeting

**Published:** 1886-04

**Authors:** 


### THE PLACE OF MEETING.

The permanent reference of the selection of the next place of meeting to the Executive Committee of the American Dental Association is a thing which may result beneficially or the reverse, according to the composition of that committee and its liability to be influenced by personal considerations. It is quite within the bounds of possibility that circumstances may arise during the year that may make it advisable to visit some place not thought of at the time of meeting, or developments may make it unwise to hold the meeting at the place which the majority of the members would have chosen. On this account it may be well to refer the choice to the committee.

But on the other hand, it might possibly be charged that some member or members had a personal end to gain in the selection of a particular place, and that their own selfish gratification outweighed their desire for the good of the Association. There has, at times, been manifested too great a desire to manipulate and manage the society, after the fashion of a political caucus, and the reference of such important matters to a committee might tend to encourage this disposition; so it is a subject that should, in the future, be carefully considered.

This year a number of places are petitioners for the meeting, and the committee, wisely we think, has referred the matter to the members for decision. The chairman of the Executive Committee has issued circulars urging the selection of Chicago, while the secretary has laid the claims of San Francisco before the members.

In this number we print the circulars issued, and lay before the profession sufficient data to enable each one intelligently to make a decision. To do this it has been found necessary to devote to this subject the space usually allotted to other matter. A number of editorial articles that were already in type, have, therefore, been set aside until there is less pressure upon our pages.

It is very earnestly urged that *every member*, or intending member, should *at once* notify the chairman or the secretary of the executive committee, in unequivocal terms, of his preference in the matter. If this be promptly done the committee can soon announce the place of meeting, and preparations can at once be begun.

When a majority of the members have expressed their preferences, that should end the matter. There should be no coercion or restraint used, but a ready compliance with the wish of the majority. The society will submit to no juggling, and he is not a good member who refuses cheerfully to accept the result of the poll.

